# Protozoan communities serve as a strong indicator of water quality in the Nile River

**DOI:** 10.1038/s41598-024-66583-z

**Published:** 2024-07-16

**Authors:** Wael S. El-Tohamy, Mohamed E. Taher, Ahmed M. Ghoneim, Russell R. Hopcroft

**Affiliations:** 1 Zoology Department, Faculty of Science, Damietta University, Damietta, Egypt; 2 College of Fisheries and Ocean Sciences, University of Alaska Fairbanks, Fairbanks, AK USA

**Keywords:** Bioindicator, Protozoa, Water quality, Nile River Damietta branch, Ecology, Zoology

## Abstract

The relationship between the protozoan communities and environmental variables was studied in the Nile River to evaluate their potential as water quality indicators. Protozoans were sampled monthly at six sampling sites in the Nile's Damietta Branch across a spatial gradient of environmental conditions during a 1-year cycle (February 2016–January 2017). The Protozoa community was comprised of 54 species belonging to six main heterotrophic Protozoa phyla. The abundance (average, 1089 ± 576.18 individuals L^−1^) and biomass (average, 86.60 ± 106.13 μg L^−1^) were comparable between sites. Ciliates comprised the majority of protozoan species richness (30 species), abundance (79.72%), and biomass (82.90%). Cluster analysis resulted in the distribution of protozoan species into three groups, with the most dominant species being the omnivorous ciliate *Paradileptus elephantinus*. Aluminium, fluoride, and turbidity negatively affected abundance and biomass, while dissolved oxygen and potassium positively impacted biomass. Of the dominant species recorded over the study area, the amoebozoa *Centropyxis aculeata* was associated with runoff variables, while the bacterivorous ciliates *Colpidium colpoda*, *Glaucoma scintillans*, and *Vorticella convallaria* were related to the abundance of heterotrophic bacteria, phytoplankton biomass, and total organic carbon. Total dissolved salts, PO_4_, NH_3,_ NO_2_, dissolved oxygen, and total organic carbon were the strongest causative factors for protozoa distribution. The α-Mesosaprobic environment at site VI confirmed a high load of agricultural runoffs compared to other sites. This study demonstrates that protozoans can be a potential bioindicator of water quality status in this subtropical freshwater river system.

## Introduction

The Nile is the longest river in the world^1^ and is considered the lifeblood of Egypt as its primary source of drinking water^2^. For thousands of years, the river has created a narrow strip of fertile land that made agriculture possible and became the backbone of Egyptian civilization^3^. Most of Egypt's population is still concentrated on the Nile River, which contributes to agriculture, industry, fishing, and transport and is central to millions living along its banks^4^. Despite the foregoing, the river is exposed to many industrial or agricultural processes that can cause severe environmental, health, and economic damage to its waters. Anthropogenic activities, along with natural processes like climatic conditions and erosion factors, change the river's physical and chemical characteristics^5^.

The spatiotemporal differences in physical and chemical properties along the river have led to different habitat types and planktonic communities specific to them^6^. Some organisms are highly sensitive to environmental disturbances, thereby serving as biological indicators when perturbations lead to changes in their community composition^7^. Thus, biological ecosystem assessment not only verifies water quality but can suggest underlying physiochemical characteristics of that environment that may be changing. The free-living heterotrophic planktonic Protozoa are pragmatic indicators of such roles.

Heterotrophic Protozoa comprise a substantial component of planktonic ecosystems, found in almost every aquatic habitat, including streams, rivers, lakes, ponds, and estuaries. They are an essential nutritional source for some metazoan–like copepods^8^—thereby transferring elements and energy from lower (e.g., algae and bacteria) to higher trophic levels^9^. Protozoa are also one of the ultimate decomposers of organic matter in nature^10^.

Protozoa have many advantages as biological indicators for assessing water quality. Foremost, their populations are highly dynamic numerically due to the rapidity of cell division, encystment, or excystment^11^. Thus, the Protozoa community structure can respond quickly to environmental change, suggesting its utility as an indicator of changes in aquatic ecosystems^11,12^. Not surprisingly, the response of each protozoan taxa is related to specific influential factors such as temperature, salinity, nutrient supply, dissolved oxygen, organic matter, eutrophication, and pollution level^13^. Several additional qualities make Protozoa effective bio-indicators. Firstly, due to their short life cycle, changes in environmental parameters quickly lead to gains or losses in their populations^14^. Secondly, their delicate cell boundaries facilitate the rapid transfer of and response to surrounding chemical stressors^15^. Thirdly, many protozoans can tolerate extreme environmental conditions, although population dynamics, including abundance and biomass, are typically impacted. These changes in abundance and biomass can be reliable indicators of the resistance of the river biological community to the effects of organic pollution^16^. Fourthly, protozoans are relatively easy to count in lab^17^. Finally, many Protozoa species appear global in their spatial distributions, each thriving wherever it encounters a specific combination of environmental conditions^18^.

The classification of Protozoa species in any reputable study should be considered, where it determines the properties of the species and facilitates their distribution in groups (phyla), each of which carries common characteristics, thus providing a better understanding of the hypothetical reasons for the relationship with other factors^19^. Therefore, accurately identifying the Protozoa species associated with the environmental factors is necessary to conduct a meaningful ecological study. Although many studies have examined planktonic Protozoa community's distribution, composition, and dynamics and their role as biological indicators in lotic systems, few have been conducted within the Nile River^18,20,21^. No studies have been conducted specifically on planktonic Protozoa in the Damietta region. Still, some Protozoa have occasionally been included as a part of the zooplankton species inventories without detailed information on their diversity, abundance, or biomass. Hence, this study has been undertaken with the objectives to (1) provide comprehensive taxonomic information about the communities of these freshwater free-living Protozoa; (2) investigate the spatial and temporal distribution of the composition, abundance, and biomass of Protozoa; (3) determine the relationships among Protozoa communities and environmental variables and (4) ascertain the applicability of Protozoa communities as bioindicators for water quality assessment.

## Material and methods

### Area of study

**Figure 1:**
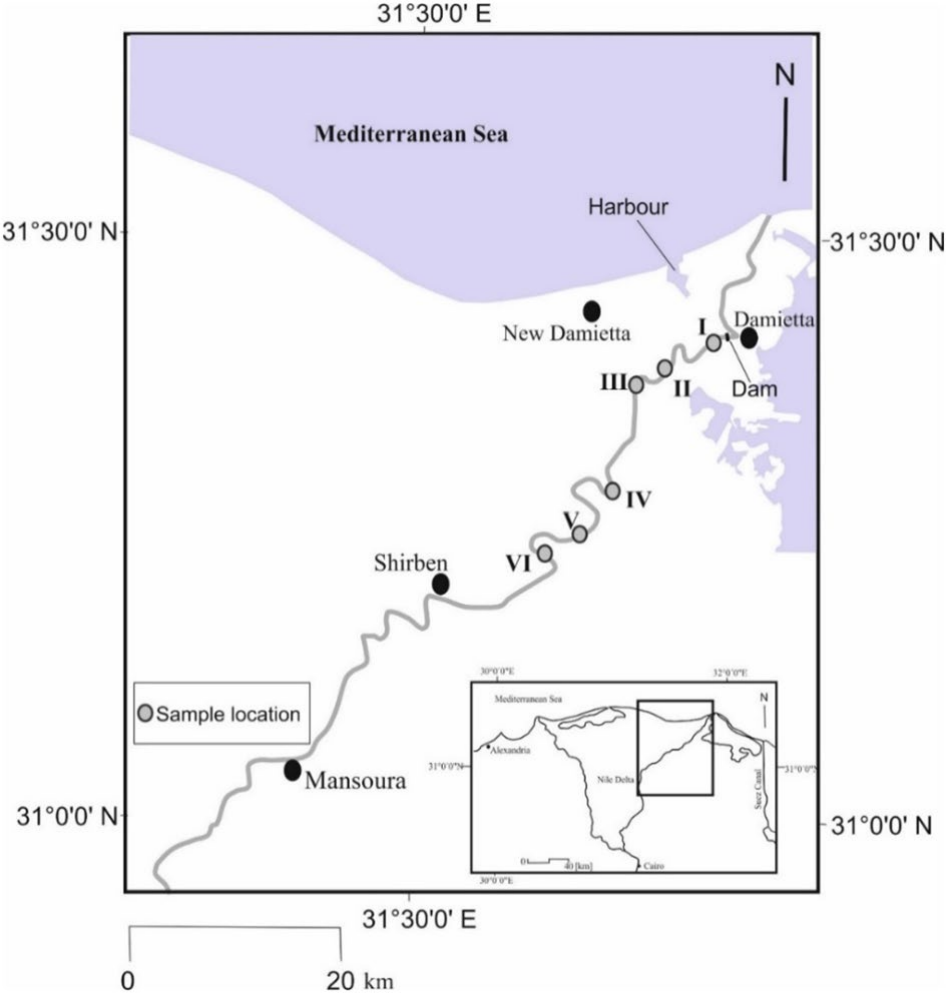
Map of the study area showing the sampling locations. This figure was generated using Esri ArcMap v. 10.4.1.

The Damietta branch of the Nile River is one of the two primary passageways through the Nile Delta. It is located just before the estuary and lies entirely within a wetland ecosystem that supports agricultural activities, where the cultivated land in the governorate constitutes about 74% of the land area. It is surrounded by urban and rural areas that expose it to anthropogenic impacts. The study was performed in the northern part of the Damietta branch within the Damietta Governorate. Six sites were selected, situated along a total waterway length of 35 kms (Fig. 1, I–VI). Sites were preferred based on settlements and agricultural activities. Site I was close to the Al-Adliya Water Treatment Plant (WTP) intake, with an average depth of 5.5 m, 2.5 km upstream of Damietta City on the river's eastern bank. Site II was near the intake of Al-Bostan WTP, with an average depth of 5.2 m, 500 m downstream of Al-Bostan village on the river's eastern bank. Site III was 4 km upstream of Kafr Soliman city on the western bank; the average depth was 4.3 m. Site IV was located in front of Al-Shenawy WTP intake on the river's eastern bank, the river island of Sharabas, an area of intensive agricultural activities 4 km upstream; the average depth was 4.7 m. Site V was 500 m downstream of Daqahla village, with an average depth of 6.6 m, and was located in an area with numerous agricultural and fishing activities. Site VI was located in an area of heavy agricultural activities from the discharge of Al-Mayasra River island effluents; the average depth was 3.1 m (Fig. 1).

### Data source

#### Samples collection

Water samples were collected monthly from February 2016 to January 2017 at six sampling sites along the Nile River Damietta Branch. A 2.2 L Van Dorn sampler was used for collecting water approximately 1 m below the river water surface. The samples were collected and manipulated according to the established protocols of the American Public Health Association (APHA)^22^.

Temperature (°C), pH, dissolved oxygen (DO), electrical conductivity (EC), and total dissolved solids (TDS) were measured in the field using portable water quality analyzers. In the laboratory, the determination of turbidity (NTU), alkalinity (CaCO_3_), total hardness, nitrite (NO_2_‾), ammonia (NH_3_^+^), silicate (SiO_3_^2−^), and phosphate (PO_4_^3−^) was done based on APHA procedures^22^. Sulphate (SO_4_^2−^) was measured using the turbidimetric method of Sheen, et al.^23^. The cations [Sodium (Na^+^), Potassium (K^+^), and Calcium (Ca_2_^+^)] and anions [Fluorides (F^-^) and Chlorides (Cl^-^)] were measured using ion chromatography (Dionex model: ICS-3000). The heavy metals, aluminium (Al), iron (Fe), manganese (Mn), cadmium (Cd), copper (Cu), nickel (Ni), lead (Pb), and zinc (Zn) were estimated by atomic absorption spectroscopy (Varian SpectrAA) following the standard acid digestion technique as described by the standard methods^22^. Also, by following APHA protocols^22^, water organic pollution was investigated using 5-day biological oxygen demand (BOD), chemical oxygen demand (COD), and total organic carbon (TOC). The phytoplankton biomass (as chlorophyll-a) was determined spectrophotometrically^24^. The abundance of heterotrophic bacteria (HPC) was determined according to APHA methods, with 100 ml of each serially diluted water sample transferred to sterilized agar plates in duplicate and incubated at 37 °C for 24–48 h.

#### Protozoa community

Protozoa samples were collected in 1000 mL amber polypropylene bottles from each sampling station. The samples were fixed with acidic iodine Lugol's solution (0.7 mL per 100 mL) and then stored in a darkened refrigerator. Samples were prepared for examination using the plankton concentration technique and pipetting method. The supernatant water was pumped out through a clear acrylic plastic tube provided with a fine nylon cloth filter fitted on its end (mesh size: 20 µm), concentrating the sample to ~ 50 mL volume. For the taxonomic enumeration of Protozoa, a volume of 1 mL aliquot of each concentrated sample was placed in the Sedgwich-Rafter counting cell, then examined under a light microscope (Optika, model: B-130) at magnifications of × 100 to × 400^25^. The number of counted individuals was calculated using the following equation^22^:

$$Number/ml= \frac{C \times {V}_{C} \times 1000}{{V}_{T}\times N}$$

where C = number of organisms counted, N = number of counted squares, while 1000 is the total number of squares, V_C_ = final volume of the concentrated sample (mL), and V_T_ = the total volume of the initial grab sample (mL).

**Figure 2:**
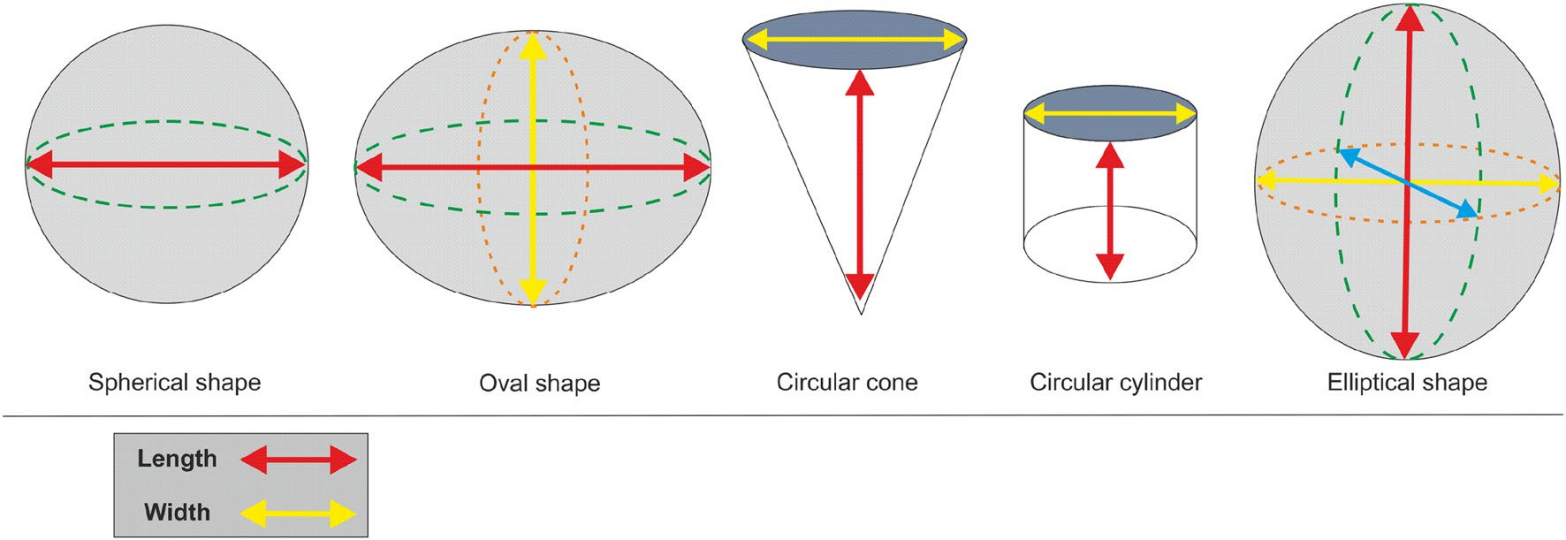
Potential geometric shapes of the Protozoa species in the study area.

Protozoa classification and identification to species levels was according to Edmondson^26^, Corliss^27^, Foissner and Berger^28^, and Patterson^29^. An ocular micrometre was used to measure the cellular dimensions (length and width as µm) for biomass calculations. Then, the biovolumes were computed as µm^3^, assuming the volumetric formula of the closest standard geometric shape (Fig. 2) as follows:

**Table:** 

Spherical shape volume = 1/6 × π × l^2^	e.g., *Glaucoma scintillans*
Oval shape volume = 1/6 × π × l × w^2^	e.g., *Didinium nasutum*
Elliptical shape volume = 1/6 × π × l × w × ½ w	e.g., *Euplotes muscicola*
Circular cone volume = 1/12 × π × l × w^2^	e.g., *Paradileptus elephantinus*
Circular cylinder volume = 1/4 × π × l × w^2^	e.g., *Strobilidium spiralis*

where l = protozoan cell length (µm), w = protozoan cell width (µm), and π (constant) = 3.14.

The calculated bio-volume was then converted to dry weight using 0.524 pg µm^−3^^30^, and then the biomass was calculated and expressed as µg L^−1^.

### Data analysis

Mean abundance (N) (individual L^−1^) and biomass (B) (μg L^−1^) were calculated for each species. The diversity of Protozoa communities at each site was described by native species richness (S) and the Shannon diversity index (H^′^)^31^. Shannon index was computed as:

$$H{\prime}= \sum_{i=1}^{s}\frac{{n}_{i}}{N}\times ln\frac{{n}_{i}}{N}$$

where *n*_*i*_ is the abundance of species *i*, *N* is the total abundance in the sample, and *ln* is the natural logarithm.

Dominance values of Protozoa species were calculated according to the equation of Xu and Chen^32^:

$$dominance= \frac{{n}_{i}}{N}{f}_{i}$$

where *f*_*i*_ is the occurrence frequency of species *i*. Considering that the dominant species was defined where the dominance value was ≥ 0.02,

Based on the distribution of a taxon with respect to the saprobic level, the saprobic index (SI) is calculated according to the Pantle and Buck^33^ formula.

$$SI=\frac{\sum Sh}{\sum h}$$

where SI = saprobic index, *S* = saprobic index of each individual species, and *h* = abundance of each species. As shown in Table 1, water quality studies classified the saprobic system into seven saprobic levels^34^.

**Table 1:** Classification of the waterbodies by the saprobic system.

Saprobic level	Saprobic index	Degree of organic pollution	Habitat quality class
Oligosaprobic	1.0 to < 1.5	Non-polluted	I
Oligosaprobic to β-Mesosaprobic	1.5 to < 1.8	Waters without organic load	I-II
β- Mesosaprobic	1.8 to < 2.3	Waters with a moderate organic load	II
β- Mesosaprobic to α- Mesosaprobic	2.3 to < 2.7	Waters with critical organic load	II-III
α- Mesosaprobic	2.7 to < 3.2	Waters strongly polluted/	III
α- Mesosaprobic to polysaprobic	3.2 to < 3.5	Waters are very strongly polluted	III-IV
Polysaprobic	3.5 to < 4.0	Waters exceptionally polluted	IV

Analysis of variance (ANOVA) was used to test the spatial and temporal differences in abundance, biomass, and diversity indices of Protozoa communities. The data were tested for normality before analysis and transformed into natural logarithms when necessary. ANOVA analysis was performed using SPSS 18. The correlation between the main indices of the Protozoa community and environmental factors was analyzed and visualized by the R packages Psych^35^.

A principal components analysis (PCA) was used to ordinate the 72 sample units and 32 environmental variables on a few factorial axes or principal components (PCs) to reduce the practical dimensionality of the dataset^36^. The correlation matrix was used to check the relationship between environmental parameters and the PCs, and the results were used to eliminate variables with a correlation <  ± 0.5 from the ordination plot. A total of 13 variables were not included (Table 2). The PCA was performed from the environmental variables' linear correlation matrix after the data's logarithmic transformation, except for the pH data.

**Table 2:** Mean value ± SD of water quality parameters taken monthly at the sampling sites during the study period.

Sampling sites	I	II	III	IV	V	VI	First two PCA axes	
							PC1	PC2
Temp. (°C)	26.97 ± 6.59	27.75 ± 6.53	26.81 ± 6.82	24.66 ± 5.95	25.02 ± 6.01	24.69 ± 5.53	**–0.64**	**–0.58**
pH	8.26 ± 0.25	8.27 ± 0.17	8.35 ± 0.22	8.23 ± 0.22	8.16 ± 0.18	8.10 ± 0.19	**–0.55**	**–**0.03
Turb. (NTU)	3.28 ± 0.88	4.26 ± 1.24	4.65 ± 1.68	4.68 ± 2.46	5.24 ± 1.83	5.96 ± 1.92	**–**0.25	**–0.50**
TDS (mgL^–1^)	274.08 ± 37.78	278.67 ± 40.93	277.90 ± 42.08	279.08 ± 46.67	278.42 ± 45.64	280.25 ± 47.88	**0.72**	0.47
EC (µsCm^–1^)	414.42 ± 61.79	428.67 ± 63.14	427.50 ± 65.01	428.42 ± 71.88	428.25 ± 70.15	429.08 ± 69.49	**0.69**	0.47
Cl^–^ (mgL^–1^)	35.50 ± 9.27	36.67 ± 9.12	36.0 ± 9.15	36.75 ± 10.06	36.33 ± 11.53	37.25 ± 10.58	**0.72**	0.43
THard. (mgL^–1^)	168.83 ± 5.22	168.17 ± 5.94	168.17 ± 4.78	170.17 ± 4.93	169.50 ± 4.91	169.17 ± 4.93	**0.63**	0.44
Ca^2+^ (mgL^–1^)	40.07 ± 0.80	39.67 ± 0.87	39.80 ± 1.14	40.13 ± 0.82	40.07 ± 0.80	40.20 ± 0.77	**–**0.02	0.14
Mg^2+^(mgL^–1^)	16.56 ± 1.27	16.56 ± 1.40	16.52 ± 1.41	16.76 ± 1.11	16.56 ± 1.13	16.48 ± 1.30	**0.62**	0.37
Alk. (mgL^–1^)	136.83 ± 17.71	140 ± 18.51	139.50 ± 19.22	139.83 ± 19.27	139.83 ± 18.63	139 ± 18.83	**0.56**	0.42
Na^+^ (mgL^–1^)	39.58 ± 5.95	39.08 ± 5.95	38.83 ± 5.78	39.33 ± 5.99	39 ± 5.74	38.83 ± 5.41	**0.68**	0.00
K^+^ (mgL^–1^)	5.70 ± 0.28	5.70 ± 0.27	5.72 ± 0.31	5.75 ± 0.28	5.72 ± 0.31	5.74 ± 0.33	**–**0.08	0.13
DO (mgL^–1^)	7.93 ± 1.38	7.85 ± 1.70	8.30 ± 1.49	8.28 ± 1.71	8.12 ± 1.66	7.83 ± 1.70	0.09	**0.55**
COD (mgL^–1^)	44.96 ± 13.26	36.57 ± 19.34	58.17 ± 46.74	41.13 ± 31.69	52.02 ± 46.83	59.79 ± 48.07	0.41	**–0.90**
BOD (mgL^–1^)	4.90 ± 2.58	6.77 ± 2.93	8.23 ± 3.91	5.08 ± 2.20	5.68 ± 3.03	8.46 ± 4.39	**–0.56**	**–**0.05
NH_3_ (µgL^–1^)	302.67 ± 133.26	291 ± 125.9	292 ± 125.36	314.17 ± 140.10	319 ± 135.17	334.08 ± 149.16	**–0.51**	0.08
NO_2_ (µgL^–1^)	20 ± 2.26	20.83 ± 3.46	19.50 ± 2.97	22.42 ± 2.35	22.33 ± 3.42	21.92 ± 2.91	**–0.51**	0.07
PO_4_ (µgL^–1^)	120 ± 8.53	139.17 ± 13.11	133.33 ± 16.14	145 ± 10	165 ± 15.67	169.17 ± 9.0	**–**0.02	0.01
SiO_3_ (µgL^–1^)	1144.20 ± 599.14	1153.30 ± 595.3	1147.50 ± 592.32	1165 ± 600.70	1209.20 ± 629.62	1229.20 ± 637.56	**–**0.04	**–**0.04
TOC (mgL^–1^)	5.49 ± 0.85	5.62 ± 0.98	5.96 ± 1.35	5.72 ± 1.10	5.55 ± 0.87	5.37 ± 1.05	**–0.53**	–0.27
SO_4_ (mgL^–1^)	29.75 ± 2.05	30.42 ± 2.91	28.83 ± 1.40	30 ± 1.48	30.08 ± 1.62	31.75 ± 1.82	–0.07	0.09
F^–^(µgL^–1^)	377.42 ± 13.44	374.58 ± 11.80	373.60 ± 13.6	373.17 ± 13.85	372.92 ± 15.70	371.92 ± 16.05	–0.23	0.18
Al (µgL^–1^)	43.33 ± 18.75	47.50 ± 10.55	49.17 ± 13.11	90.81 ± 14.97	50 ± 23.35	65 ± 21.95	0.09	–0.12
Fe (µgL^–1^)	34.67 ± 22.03	45.75 ± 28.42	46.08 ± 45.02	32.50 ± 33.34	55.75 ± 41.44	75.88 ± 56.32	–0.07	–0.05
Mn (µgL^–1^)	25.50 ± 16.67	29.67 ± 21.47	30 ± 29.60	23.58 ± 13.98	26.83 ± 14.12	34.17 ± 22.11	–**0.51**	–0.36
Cd (µgL^–1^)	0.04 ± 0.04	0.05 ± 0.04	0.04 ± 0.05	0.12 ± 0.04	0.12 ± 0.06	0.05 ± 0.05	0.05	–0.15
Cu (µgL^–1^)	0.42 ± 1.44	1 ± 3.16	2.67 ± 4.08	0.17 ± 0.58	0.50 ± 1.45	2.08 ± 4.14	–0.22	0.00
Ni (µgL^–1^)	1.69 ± 0.88	1.08 ± 1.45	0.52 ± 0.21	0.93 ± 1.40	0.53 ± 0.29	0.57 ± 0.26	0.10	–0.04
Pb (µgL^–1^)	0.40 ± 0.28	0.31 ± 0.21	0.34 ± 0.24	1.22 ± 0.22	0.31 ± 0.21	0.63 ± 0.25	–0.12	–0.07
Zn (µgL^–1^)	3.31 ± 4.89	2.48 ± 4.05	1.07 ± 1.66	4.93 ± 6.51	1.71 ± 3.90	1.45 ± 3.34	–0.10	0.30
Chl-a (µgL^–1^)	7.11 ± 4.80	6.21 ± 2.98	7.64 ± 3.25	7.49 ± 3.09	5.91 ± 2.28	5.29 ± 2.82	**–0.57**	–0.29
HPC (CFU 1mL^–1^)	506.67 ± 379.77	645 ± 505.60	615.83 ± 358.29	802.50 ± 627	590.83 ± 465.28	651.67 ± 453.41	**–0.86**	–0.16

Correlation coefficients between environmental variables and the first two PCA axes are included. Stronger PCA correlations are indicated in boldface.

Temperature (temp), Turbidity (Turb), Total hardness (T. Hard), Alkalinity (Alk), Chlorophyll-a (Chl-a), Heterotrophic bacteria (HPC).

A multivariate ordination analysis was applied to determine the environmental factors that best explained the structure and distribution of Protozoa species. Before analysis, the abundance of Protozoa species was square-root transformed to reduce the effect of high densities^37^. A preliminary detrended correspondence analysis (DCA) was performed to estimate the gradient length of the first axis in standard deviation (SD) units for specifying the unimodal or linear response of the primary data^38^. Gradient length was longer than 3 (7.95 SD), indicating unimodal response, concluding in the Canonical Correspondence Analysis (CCA) as the appropriate multivariate regression analysis^39^. Prior to the CCA analysis, the stepwise regression analysis was applied to identify variance inflation factors (VIF). VIF analysis is a common way for detecting useless constraints; a general rule is that the large value of the Variance Inflation Factor (VIF > 20) means that a given variable is strongly correlated and, therefore, has no unique contribution to the regression equation^40^. VIF analysis results were used to eliminate selected variables from further analyses. CANOCO for Windows 4.5^40^ was used for the PCA and CCA analysis. Ward's clustering method was carried out using the Euclidean distances^41^ and applied to square-root transformed abundance values of Protozoa species using the StatSoft Statistica 8.0 software package. The biota-environment correlation was tested using the routine RELATE and BIOENV (biota-environment correlation analysis), which were analyzed using the PRIMER v6.1 package to explore potential relations between Protozoa community structure and the abiotic data.

## Results and discussion

### Environmental status

The environmental variables of the six sampling sites over the 12 months were summarized in Table 2. Many parameters like temperature, pH, TDS, EC, chlorides, total hardness, calcium, magnesium, sodium, and potassium showed minor differences at all sampling sites. Turbidity measurements averaged 4.68 ± 1.88 NTU; the most turbid sites were V and VI. The concentrations of DO were usually higher than 7.50 mgl^-1^ at all sites, with the minimum average value at site VI (7.83 mgl^-1^) and the maximum at site III (8.30 mgl^-1^). EC ranged from 335 to 544 μsCm^-1^, and TDS ranged from 218 to 364 mgl^-1^; both exhibited the same spatial and temporal patterns reflecting a direct linear relation between each^42^; site IV was the highest with average values of 429.42 μsCm^−1^ and 280.25 mgl^−1^, respectively. BOD and COD ranged from 0.70 to 17 and 3.50 to 177.70 mgl^−1^, respectively. The high levels of BOD and COD induced a concern that organic pollution may threaten the water quality in the Nile River. The ranges of dissolved nutrients, NH_3_^+^-N, were 24–480 µgl^−1^, while NO_2_^−^–N, which exhibits an unstable oxidation state between the reduction of nitrates and oxidation of ammonia^43^, were 16–28 µgl^-1^. PO_4_^3−^-P ranged between 110 and 180 µgl^−1^. SiO_3_^2−^ which may be consumed by testate amoebae for reproduction^44^, ranged between 1120 and 1260 µgl^−1^. Site VI was the most exposed to these chemicals, mainly attributed to agricultural runoff from river islands. The temperature averaged 25.98 ± 6.16, illustrating seasonal variation as the minimum value was in January 14.50 °C and the maximum value was in August 36.60 °C, considering its significant effect on most physical and chemical properties of water systems^45^, and its influence on many biological processes as metabolic rate and photosynthesis^46^. Some parameters like pH, nutrients, BOD, HPC, and Chlorophyll-a demonstrated seasonal variations along with temperature, reaching their maximal levels during summer; however, winter months recorded their minimal levels. Heavy metals resemble severe environmental problems due to their toxic nature as well as their tendency to accumulate in living cells^47^; metals including Cd, Pb, Ni, and Zn ranged from 0.01–0.20, 0.06–1.86, 0.06–5.37 and 0.2–20 µgl^−1^, respectively.

**Figure 3:**
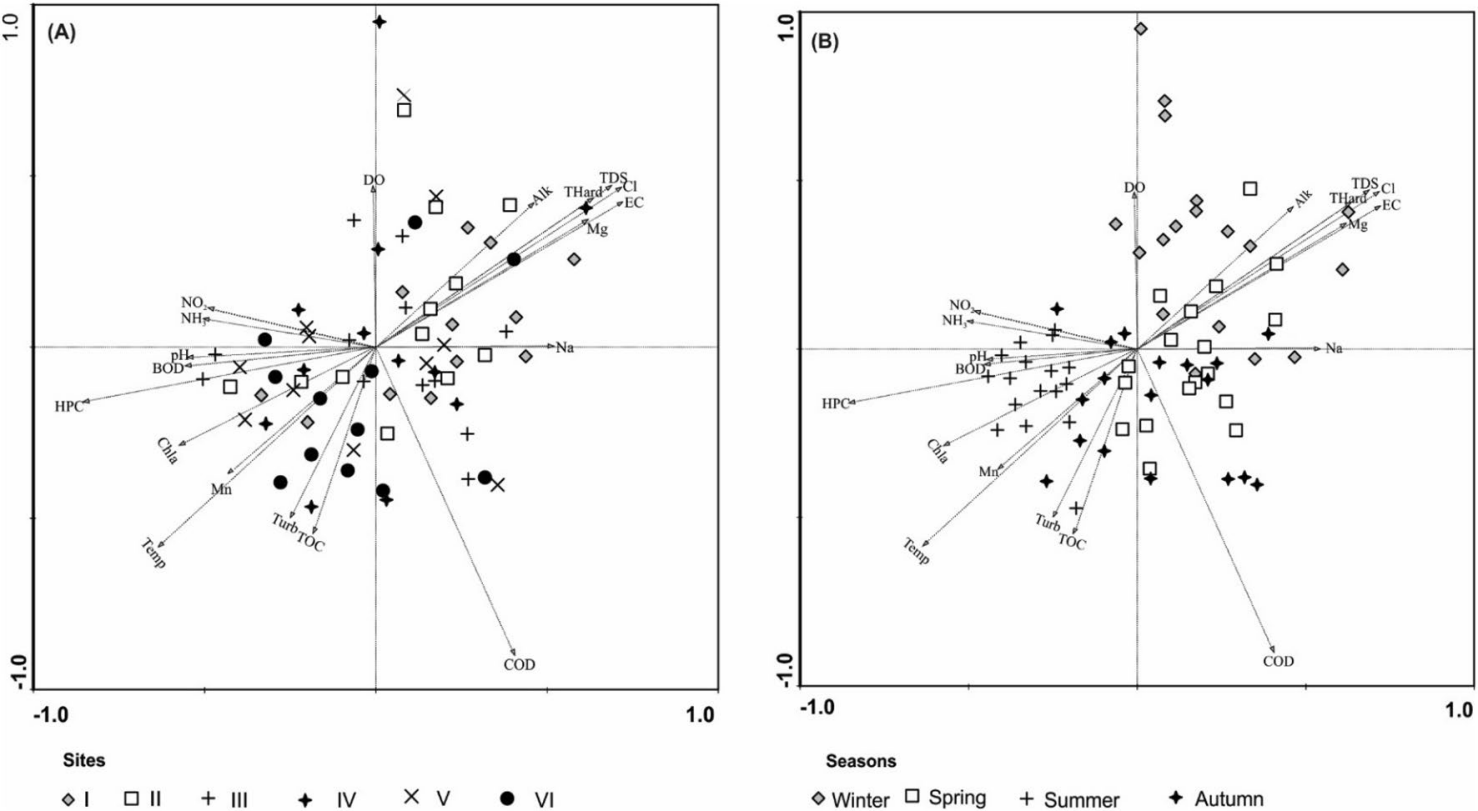
Ordination diagram by PCA of the 72 sampling units classified spatially (**A**) and seasonally (**B**), based on the correlation matrix of the most important environmental parameters. As shown in Table 2, variables with a correlation <  ± 0.5 were eliminated from the ordination plot.

The spatial and temporal data for environmental variables were analyzed and described using Principal Component Analysis (PCA) (Fig. 3). The first two PCA axes contributed up to 62.70% of the total variance in environmental variables. It can be observed in the ordination diagram (Fig. 3) that most winter sample units are positively correlated with the first axis with high concentrations of dissolved salts related parameters of EC, TDS, Cl, Na, and Mg, along with hardness and alkalinity, mostly at samples units of sites I, II, and III. Damietta region exhibits a highly seasonal rainfall pattern, having an annual rainfall of around 105.60 mm, with the maximum amount occurring during winter^48^. High concentrations of dissolved salt parameters in the rainy seasons were mainly due to high rain that increased rain-splash and runoff detachment, which caused surface soil erosion into the river system. NH_3_, NO_2_, HPC, BOD, and chlorophyll-a runoff parameters were more associated with elevated temperature during summer, which was characterized by increasing anthropogenic activities^5^, such as agriculture, particularly at sites V and VI. High turbidity, TOC, and COD levels related to the second axis showed a temporal association with most spring and autumn sample units, with no specific spatial association. The BOD vector was shorter and far from that of COD, thus indicating the presence of organic compounds that are difficult to biodegrade or that some heavy metals may exist and destroy the microorganisms^49^. The high average COD concentrations during late spring and autumn were likely due to increased organic matter available through soil microbiological decomposition and leaf fall^50^. The combination of COD and carbon (TOC) vectors in the ordination diagram along with spring and autumn sample units, supports our results.

The application of PCA has allowed an analysis of the possible correlations between the important parameters. For the study area, the observed patterns suggest that the water composition varied considerably along the river length during summer, autumn, and late spring, probably because of different anthropogenic activities near the riverbanks. A more stable water composition was observed during the low-activity winter months. This may imply that water quality along the river length is a periodic phenomenon influenced by human activities near the riverbanks throughout the year.

### Protozoa community dynamics

#### Taxnomic structure, species distribution, and variations in species number, abundance, diversity, and biomass

The free-living planktonic Protozoa community in this freshwater study area was represented by 54 species (Table 4), a value well within the range found in most lotic environments of 20–200 species^14^. The species identified belonged to 45 genera that were more diversified than reported for other regions along the Damietta branch (e.g., Qalyubia and Daqahlia, 35 genera recorded) but similar to that of the Menofia region on the Nile's Rosetta branch with 45 genera^20^. The Damietta protozoan community consisted of six main phyla: Amoebozoa (10 species), Choanozoa (4 species), Heliozoa (4 species), Cercozoa (5 species), Myzozoa (1 species), and Ciliophora (30 species). The ciliate community was the most predominant, which may be a consequence of ciliates' ability to tolerate extremes of environmental conditions^51^. The prevalence of ciliates was consistent with numerous studies in Egypt, e.g., Refs.^20,52,53^, and similar subtropical settings, e.g., Refs.^11,54^. Autumn and winter were more diverse (28 and 29 species, respectively) than spring and summer, with 24 and 22 species. This temporal variation was likely due to the influence of climate changes on physical, chemical, and biological variables^55^. Spatially, there were significant differences in protozoan species number (*P* < *0.01*); site III harboured the highest species number (30 species), and the lowest (20 & 22 species) appeared at site V and site VI, respectively. In contrast, sites I, II, and IV showed relative stability in species number (26–28 species) (Table 3), reflecting a degree of similarity in geographical and physicochemical conditions between such sites^56^.

**Table 3:** Seasonal and spatial variations of Protozoa Shannon index, species richness, abundance (individual l^−1^), and biomass (µg l^−1^) in the Nile River's Damietta region.

	Shannon	Richness	Abundance	Biomass
Winter	2.83	29	888.89 ± 366.04	146.82 ± 161.63^a^
Spring	2.50	24	1155.56 ± 442.22	41.73 ± 38.51^b^
Summer	2.21	22	1222.22 ± 829.99	57.88 ± 55.29^b^
Autumn	2.70	28	1088.89 ± 549.75	100.02 ± 96.65^ab^
Site I	2.83^a^	27^ab^	983.33 ± 496.96^ab^	52.77 ± 44.39
Site II	2.71^a^	28^ab^	1166.70 ± 499.70^ab^	83.19 ± 63.32
Site III	2.79^a^	31^a^	1458.30 ± 594.61^a^	165.70 ± 177.82
Site IV	2.80^a^	26^ab^	1350 ± 648.78^a^	87.61 ± 92.41
Site V	2.52^b^	20^b^	966.67 ± 492.37^ab^	83.18 ± 116.07
Site VI	2.65^ab^	22^b^	608.33 ± 344.90^b^	47.22 ± 57.41
Community figures	3.13	54	1089 ± 576.18	86.61 ± 106.13
ANOVA				
Seasons	p˃0.05	p˃0.05	p˃0.05	p˂0.05
Sites	p˂0.05	p˂ 0.01	p˂ 0.01	P˃0.05

The letters indicate significant differences based on a one-way ANOVA analysis with Tukey's b test, where a > b.

**Figure 4:**
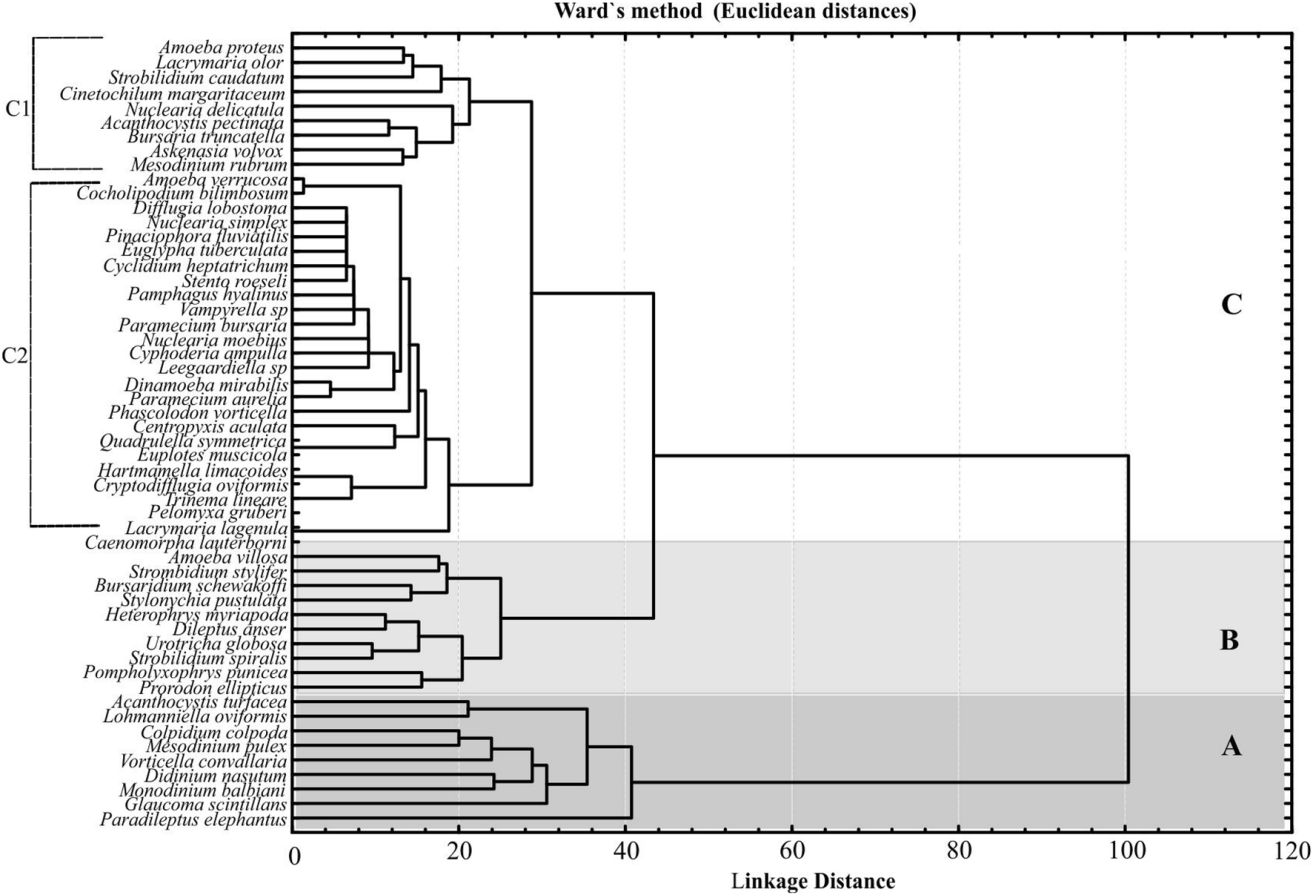
Cluster analysis of Protozoa taxa based on species abundance at the 72 sampling units, formed by Ward's method as linkage rule and Euclidean distances as similarity measure.

A dendrogram of the species distribution was plotted using the Euclidean distances on Ward's method for the square-root transformed species abundance data within the Protozoa community from the six sampling sites (Fig. 4). The cluster analysis resulted in the 54 protozoan taxa falling into three main groups at a 43% similarity level: group A was composed of 9 dominant (with relative abundance above 4%) Protozoa species contributing up to 60% in community structure at all six sites. Cluster B comprised 11 species with relatively higher frequencies, contributing up to 17% of community abundance. Cluster C included the remaining 35 species divided into two sub-clusters; the first (C1) comprised nine species that had average frequencies, and the second one (C2) was mixed and poorly separated sub-cluster, indicating the gradual changes in the community structure by the gain and loss of the different species throughout the study area. Seven species of ciliates, namely *Colpidium colpoda*, *Glaucoma scintillans*, *Vorticella convallaria*, *Didinium nasutum*, *Monodinium balbiani*, *Mesodinium pulex,* and *Paradileptus elephantinus* in group A appeared at most sampling sites, contributed 56.5% of the total Protozoa abundance, a typical pattern where a few species make up the majority of individuals^57^. Other taxa were prominent at only a few sites, such as the to heliozoan *Acanthocystis turfacea* at Site I and amoebozoan *Centropyxis aculeata* at Site VI. *Paradileptus elephantinus* was the only dominant species at all sampling sites throughout the year.

The abundance of total protozoans experienced a wide variation range (300 –2900 individuals L^-1^) with an annual average of 1089 ± 576.18 individuals L^-1^. The significant changes in protozoan population abundance could indicate flow rate variations as well as chemical changes in habitat caused by the heterogeneity of anthropogenic influence^20^. Such spatial differences were highly significant (*P* < *0.01*), where site VI harboured the lowest abundance with an average value of 608 ± 344.90 individuals L^-1^, while site III was the highest (1458 ± 594.61 individuals L^-1^). Seasonally, the abundance distribution was opposite to richness; summer had the lowest richness but the highest abundance, while winter had the highest richness but the lowest average abundance (Table 3). Enhanced summer and spring Protozoa populations agree with numerous studies, e.g., Refs.^20,58^. With respect to biomass, the values of the total community ranged between 3.60 and 588.30 µg L^−1^, with a mean biomass value of 86.61 ± 106.13 µg L^−1^. As shown in Table 3, the total Protozoa biomass showed similarity in pattern to abundance; the maximum biomass was recorded at site III that was significantly more than other sites, with an average value of 165.70 ± 177.82 µg L^−1^, while the minimum appeared at site VI, with an average value of 47.20 ± 57.41 µg L^−1^. On the seasonal scale, it is noted that for the seasons that showed an increase in abundance, their biomass was relatively small, and vice versa (Table 3), reflecting the role of observed cell sizes in the study samples^59^. It may also be that higher trophic organisms selectively consumed the large-sized Protozoa while the small-sized Protozoa remained in the environment^60^. Nonetheless, a relative correlation between biomass and abundance can be detected between sampling sites, confirming the crucial role of biomass as a measure of abundance depending on the environmental features of each site^61^.

The Shannon Weaver diversity index for the protozoan community fluctuated widely between 0.50 and 2.01, with an absolute value of 3.13. Significant differences existed in the values of the Shannon–Weaver index between different sites (*P* < *0.05*). The variation in diversity may be attributed to environmental disturbances^62^, the maximum value of which was 2.83 at site I, while site V showed a minimum value of 2.52 (Table 3). On the seasonal scale, excluding the minimal and maximal values, the diversity index showed narrow seasonal variations between the sampling sites. The relatively low diversity during summer may be attributed to the extreme values of some ecological parameters such as temperature, NO_2_, SO_4_, Cu and HPC, indicating an infrequency of species that could withstand the extremes of such environmental conditions^63^.

**Figure 5:**
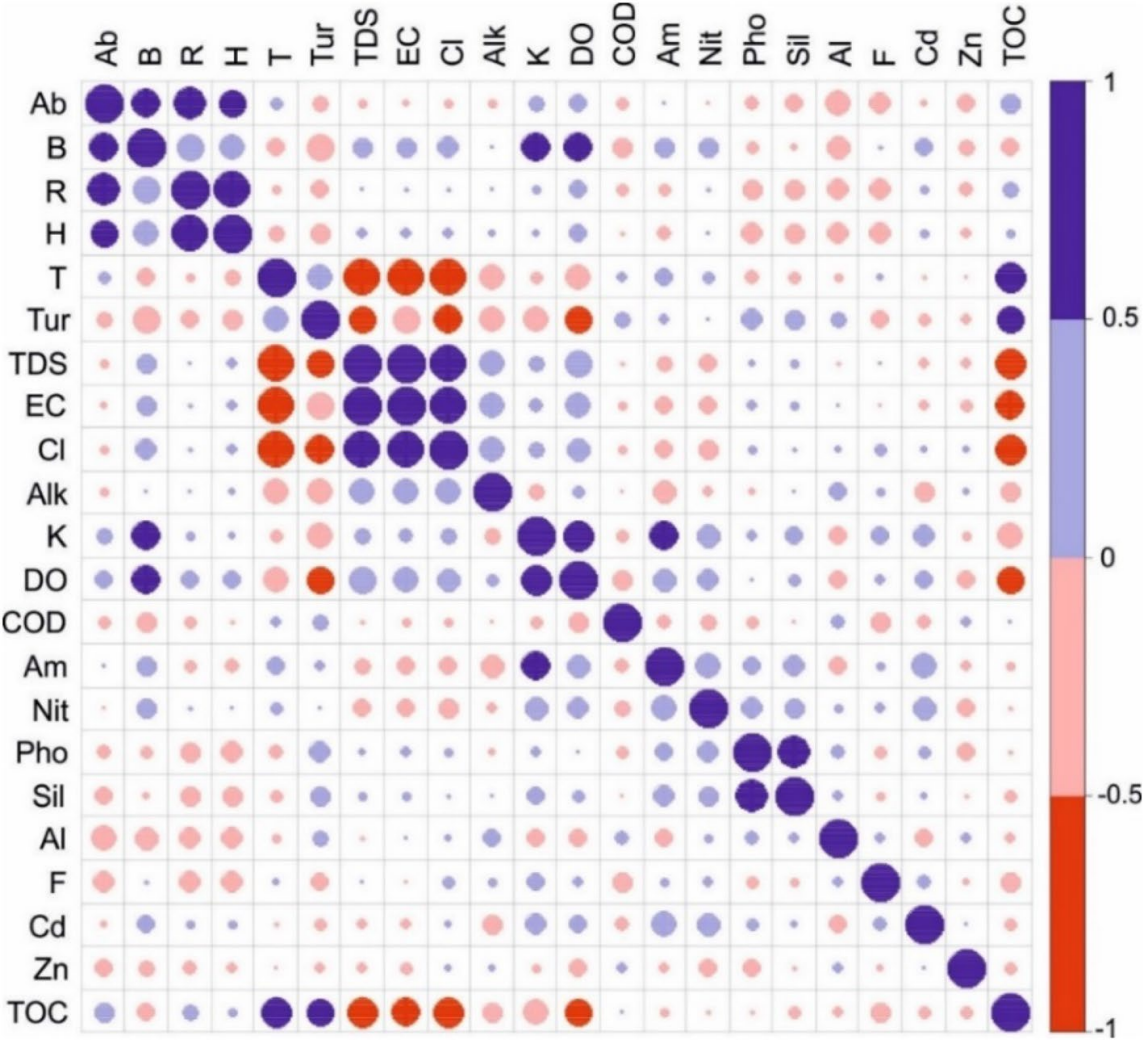
Spearman correlations between water quality parameters and Protozoa community dynamics. Only parameters with significant correlations are included in the diagram. T (temperature), Tu (turbidity), Alk (alkalinity), Am (ammonia), nit (nitrite), Pho (phosphate), Sil (silicate), TOC (total organic carbon), Ab (Protozoa abundance), B (biomass), S (species number), and H (Shannon index).

Many physicochemical parameters set up conditions that could be limiting for Protozoa (Fig. 5). TOC, which could be interpreted as the presence of bacteria or organic carbon sources in the Nile water, showed a positive correlation with total abundance (*r = 0.292, P * < *0.05* ). Dissolved oxygen showed a strong positive correlation with biomass, while the temperature was negatively correlated. According to Macek, et al.^64^, the initial biomass gradient appeared to be followed by that of the dissolved oxygen, while metabolic rates are positively correlated to temperature, which reduces the cell's biomass^65^. Also, the positive correlation between biomass and nitrogenous nutrients (NH_3_^+^ & NO_2_^−^) is a common pattern for planktonic communities^66^. Some essential elements, such as potassium and zinc, showed a strong positive correlation with biomass; according to Crawford, et al.^67^, zinc may be responsible for Protozoa cell growth stimulation. Total dissolved salts and related parameters, such as chlorides and conductivity, would positively affect community biomass, an observation similar to that of Nosek and Bereczky^68^. We suggest that increased turbidity indirectly affects the Protozoa community's biomass, richness, and diversity because it impedes filtering and consumption rates^69^. Also, the significant negative correlation of species richness and diversity with phosphate and silica levels may explain the association of such conditions with agricultural land discharges^70^. Aluminium is toxic in aquatic environments^71^, consistent with the observed adverse effects on community richness (*r* = *− 0.322, P* < *0.01*), diversity (*r* = *− 0.298, P* < *0.01*), abundance (*r* = *− 0.418, P* < *0.001*), and biomass (*r* = *− 0.403, P* < *0.001*) of the Protozoa community. According to Sauvant, et al.^72^, protists can incorporate aluminium compounds by passive diffusion through the cellular membrane or by an active process, such as phagocytosis. This physiological feature can explain the higher Protozoa uptake of aluminium in aquatic habitats. These aluminium compounds stimulated the phagocytosis rate that enhanced the aluminium bioconcentration, which induced an increased toxicity^73^. Abundance was also negatively affected by the toxicity of fluorides in the study area (*r* = *− 0.302, P* < *0.01*), a toxicity that also affected the richness and diversity index of the Protozoa community species^74^.

#### Spatial variations in abundance and biomass of protozoan groups

**Figure 6:**
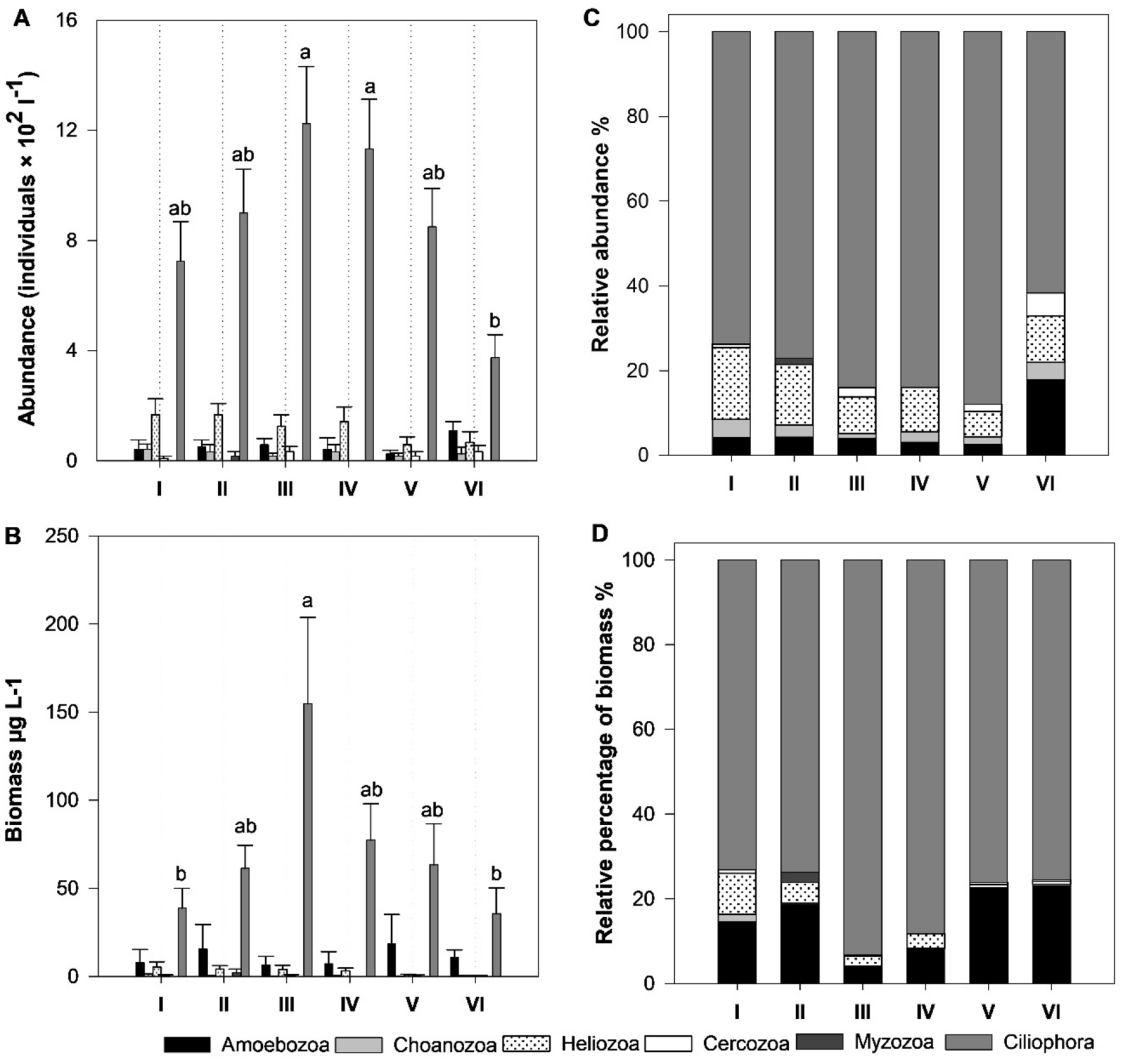
Spatial variations in abundance(**A**), biomass (**B**), relative abundance (**C**) and relative biomass (**D**) of protozoans from the six sites in the Nile River's Damietta region. The letters indicate significant differences based on one-way ANOVA with the Tuckey's test. Error bars represent the standard error of the mean.

There was a considerable difference in the relative and absolute abundance among the six main phyla (Fig. 6); phylum Ciliophora was dominant (79.72%) with an average of 868 ± 594 individuals L^−1^, followed by phyla Heliozoa (121 ± 157.40 individuals L^−1^, 11.10%) and Amoebozoa (54 ± 103.40 individuals L^−1^, 4.97%). The high contribution of Ciliophora is likely due to the advantageous characteristics of ciliates, including high reproduction rate and diversity of omnivorous species (Table 4)^14,75^. The average abundance of the phyla Choanozoa, Cercozoa, and Myzozoa were 28 (2.55%), 15 (1.40%), and 3 individuals L^−1^ (0.26%), respectively. Maximum relative abundance of ciliates (88%) was found at site V, followed by sites III and IV (84%); this reduced upstream and was minimal at site VI (62%). Relative abundances of amoebozoans (18%) and cercozoans (5%) were maximum at site VI. Helizoans (17%) were maximum at site I. The spatiotemporal and habitat type differences were among the most critical factors influencing species abundance patterns^6^. Likewise, the relative biomass of Protozoa groups showed similar patterns to abundance (Fig. 6), with some differences resulting from the variation in cellular sizes between species. Phylum Ciliophora had the highest biomass, averaging 71.80 ± 93.96 µg L^−1^ (82.9%) and an average length of 85.50 µm. Amoebozoa (the third most abundant) biomass ranked second, trading rank with the Heliozoa (the second most abundant group) because Amoebozoa individuals were larger (average length = 90 µm) than that of Heliozoa (average length = 52.40 µm). Amoebozoa biomass averaged 11.04 µg L^−1^ (12.74%), while Heliozoa averaged 2.85 µg L^−1^ (3.29%). Notably, the relative biomass of Ciliophora, and Heliozoa in particular, decreased in sites V and VI, with an increase of that of Amoebozoa, consistent with the decrease in water quality (Fig. 6).

**Table 4:** List of Protozoa species recorded at the six sampling sites, including average abundance (individuals L^−1^) and average biomass (μg L^−1^ in parentheses).

Taxa	S	Mf	Site I	Site II	Site III	Site IV	Site V	Site VI	Average
Phylum: Amoebozoa									
* Amoeba proteus*	b		8.33 (0.08)	33.33 (1.57)	25 (1.51)		8.33 (0.28)	8.33 (0.37)	13.89 (0.64)
* Amoeba verrucosa*						33.33 (5.15)			5.56 (0.86)
* Amoeba villosa*	a-b		33.33 (7.60)	16.67 (13.93)	16.67 (4.95)		16.67 (18.36)	33.33 (4.33)	19.44 (8.20)
* Hartmannella limacoides*					8.33 (0.01)				1.39 (0.0017)
* Centropyxis aculeata*	b							**41.67 (2.10)**	6.94 (0.35)
* Cocholipodium bilimbosum*						8.33 (1.85)			1.39 (0.31)
* Cryptodifflugia oviformis*					8.33 (0.01)				1.39 (0.0017)
* Difflugia lobostoma*	a-b							8.33 (0.24)	1.39 (0.04)
* Pelomyxa gruberi*	p-i							8.33 (3.43)	1.39 (0.57)
* Quadrulella symmetrica*								8.33 (0.43)	1.39 (0.07)
Phylum: Choanozoa									
* Nuclearia delicatula*			33.33 (0.22)	25 (0.17)	16.67 (0.16)	25 (0.21)	8.33 (0.06)	25.0 (0.19)	22.22 (0.17)
* Nuclearia moebius*			8.33 (0.72)	8.33 (0.11)					2.78 (0.14)
* Nuclearia simplex*						8.33 (0.04)			1.39 (0.01)
* Pinaciophora fluviatilis*							8.33 (0.06)		1.39 (0.01)
Phylum: Heliozoa									
* Acanthocystis pectinata*			8.33 (0.96)	8.33 (0.63)	16.67 (1.26)	8.33 (0.96)	8.33 (0.46)		8.33 (0.71)
* Acanthocystis turfacea*			**83.33 (0.18)**	66.67 (0.15)	75 (0.16)	83.33 (0.18)	41.67 (0.09)	58.33 (0.13)	68.06 (0.15)
* Heterophrys myriapoda*			50 (3.61)	33.33 (2.44)	25 (2.32)	16.67 (1.45)			20.83 (1.64)
* Pompholyxophrys punicea*			25 (0.35)	58.33 (0.86)	8.33 (0.15)	33.33 (0.45)	8.33 (0.15)		23.61 (0.35)
Phylum: Cercozoa									
* Cyphoderia ampulla*					16.67 (0.51)				2.78 (0.09)
* Euglypha tuberculata*			8.33 (0.43)						1.39 (0.07)
* Pamphagus hyalinus*								16.67 (0.12)	2.78 (0.02)
* Trinema lineare*					16.67 (0.05)		16.67 (0.41)		5.56 (0.08)
* Vampyrella sp.*								16.67 (0.09)	2.78 (0.01)
Phylum: Myzozoa									
* Dinamoeba mirabilis*				16.67 (2.06)					2.78 (0.34)
Phylum: Ciliophora									
* Askenasia volvox*	b	Al, Ki	16.67 (0.57)	8.33 (0.10)	8.33 (0.96)	8.33 (0.90)			6.94 (0.42)
* Bursaria truncatella*	a-b	O			50 (26.94)				8.33 (4.49)
* Bursaridium schewakoffi*					66.67 (22.03)	66.67 (16.37)	66.67 (26.55)	41.67 (17.39)	40.28 (13.72)
* Caenomorpha lauterborni*	p-m	Ba, Sb						8.33 (0.15)	1.39 (0.03)
* Cinetochilum margaritaceum*	p-i	Ba, Al			41.67 (0.06)	8.33 (0.01)	33.33 (0.04)	16.67 (0.02)	16.67 (0.02)
* Colpidium colpoda*	p-i	Ba, Fl, Al		8.33 (0.66)	**100.0 (5.86)**	**133.30 (12.65)**	16.67 (1.48)	83.33 (6.23)	56.94 (4.48)
* Cyclidium heptatrichum*	b	Ba			8.33 (0.03)				1.39 (0.005)
* Didinium nasutum*	a-b	R	50 (6.02)	41.67 (4.64)	**66.67 (9.01)**	**108.30 (8.93)**	**233.30 (13.20)**	25.0 (1.20)	**87.50 (7.17)**
* Dileptus anser*			16.67 (0.55)	16.67 (2.83)		8.33 (0.10)			6.94 (0.58)
* Euplotes muscicola*							8.33 (1.43)		1.39 (0.24)
* Glaucoma scintillans*	p-a	Ba	**66.67 (6.75)**	41.67 (2.12)	16.67 (0.12)	50 (2.50)		**125 (25.62)**	50 (6.18)
* Lacrymaria lagenula*								8.33 (0.43)	1.39 (0.07)
* Lacrymaria olor*	b	R	8.33 (0.56)	33.33 (7.32)	33.33 (1.45)				12.50 (1.55)
* Leegaardiella sp.*			8.33 (0.43)		8.33 (0.40)				2.78 (0.14)
* Lohmanniella oviformis*			8.33 (0.06)			41.67 (0.22)	**83.33 (0.37)**	**66.67 (0.33)**	33.33 (0.16)
* Mesodinium pulex*	b	O	16.67 (0.20)	33.33 (0.49)	16.67 (0.29)	**125 (1.49)**	**91.67 (1.10)**	16.67 (0.20)	50 (0.63)
* Mesodinium rubrum*			33.33 (0.16)	16.67 (0.07)	25 (0.07)				12.50 (0.05)
* Monodinium balbiani*	o-a	R	**133.3 (5.04)**	33.33 (1.09)	58.33 (1.78)	**66.67 (2.69)**	**75 (2.57)**		61.11 (2.23)
* Paradileptus elephantinus*	b	O	**175 (10.99)**	**283.30 (30.65)**	**358.30 (48.4)**	**258.33 (22.37)**	**125 (9.10)**	**75 (5.04)**	**212.50 (21.09)**
* Paramecium aurelia*	a-b	Ba	8.33 (0.11)	16.67 (0.55)					4.17 (0.11)
* Paramecium bursaria*	a-b	Ba, Al, Ki						16.67 (2.13)	2.78 (0.35)
* Phascolodon vorticella*	a-b	Al, Ki	33.33 (0.80)	16.67 (0.51)	16.67 (0.40)				11.11 (0.29)
* Prorodon ellipticus*	a-b	R		58.33 (5.47)	50 (8.08)	25 (0.81)			22.22 (2.39)
* Stentor roeseli*	a-b	O		8.33 (0.12)					1.39 (0.02)
* Strobilidium caudatum*	o-b	Ki, Al, Ba	16.67 (0.16)	8.33 (0.58)	16.67 (0.25)	16.67 (0.05)			12.50 (0.18)
* Strobilidium spiralis*			8.33 (0.11)	8.33 (0.11)		16.67 (0.29)			5.56 (0.08)
* Strombidium stylifer*						25 (1.71)	33.33 (1.05)	33.33 (0.58)	15.28 (0.56)
* Stylonychia pustulata*	b	O	25 (5.45)	16.67 (1.83)	8.33 (1.29)	58.33 (5.53)	33.33 (6.24)		23.61 (3.39)
* Urotricha globosa*	a-b	Ba, Al, Fl	8.33 (0.02)	8.33 (0.06)	16.67 (0.22)	16.67 (0.29)			8.33 (0.10)
* Vorticella convallaria*	a	Ba	41.67 (0.42)	**208.33 (2.13)**	**183.33 (1.90)**	**100 (1.19)**	50 (0.68)		97.22 (1.05)

Bold squares highlight the dominant species, saprobity levels (S), and main food source (Mf).

### Interaction between Protozoa species and environmental factors

Multivariate techniques are often more sensitive than univariate measures when exploring complicated biotic and abiotic data changes. They are valuable in revealing a relationship between community structure and habitat and identifying the main variables affecting Protozoa communities when a large set of environmental variables has been conjointly collected^76–78^. RELATE analysis revealed a significant correlation between variations in Protozoa species abundances and changes in environmental variables (*r = 0.302*, *P = 0.001*). The correlations between Protozoa abundances and environmental variables were established by multivariate biota-environment (BIOENV) analysis (Table 5). The BIOENV analysis is a potential approach to selecting environmental parameters that best explain changes in the distribution of Protozoa species. The BIOENV analysis suggests that fit continues to improve modestly through the inclusion of 7 or 8 parameters but showed no improvement with additional variables. The best models included environmental parameters TDS, PO_4_, NH_3_, NO_2_, DO, BOD, TOC, and either Al or Fe, which are most related to spatial variations of Protozoa distribution in the Nile water.

**Table 5:** Summary of Biota-environment (BIOENV) analysis results, with the two best 7 and 8 parameter correlations corresponding to different variables.

R-value	Environmental variables
0.359	TDS, PO_4_, Cl, NH_3_, NO_2_, DO, BOD, TOC
0.356	TDS, PO_4_, NH_3_, NO_2_, Fe, DO, BOD, TOC
0.347	TDS, PO_4_, NH_3_, NO_2_, DO, TOC, HPC
0.346	TDS, PO_4_, Cl, NH_3_, NO_2_, DO, TOC

R-value: Spearman correlation coefficient; for abbreviations, see Table 2.

**Figure 7:**
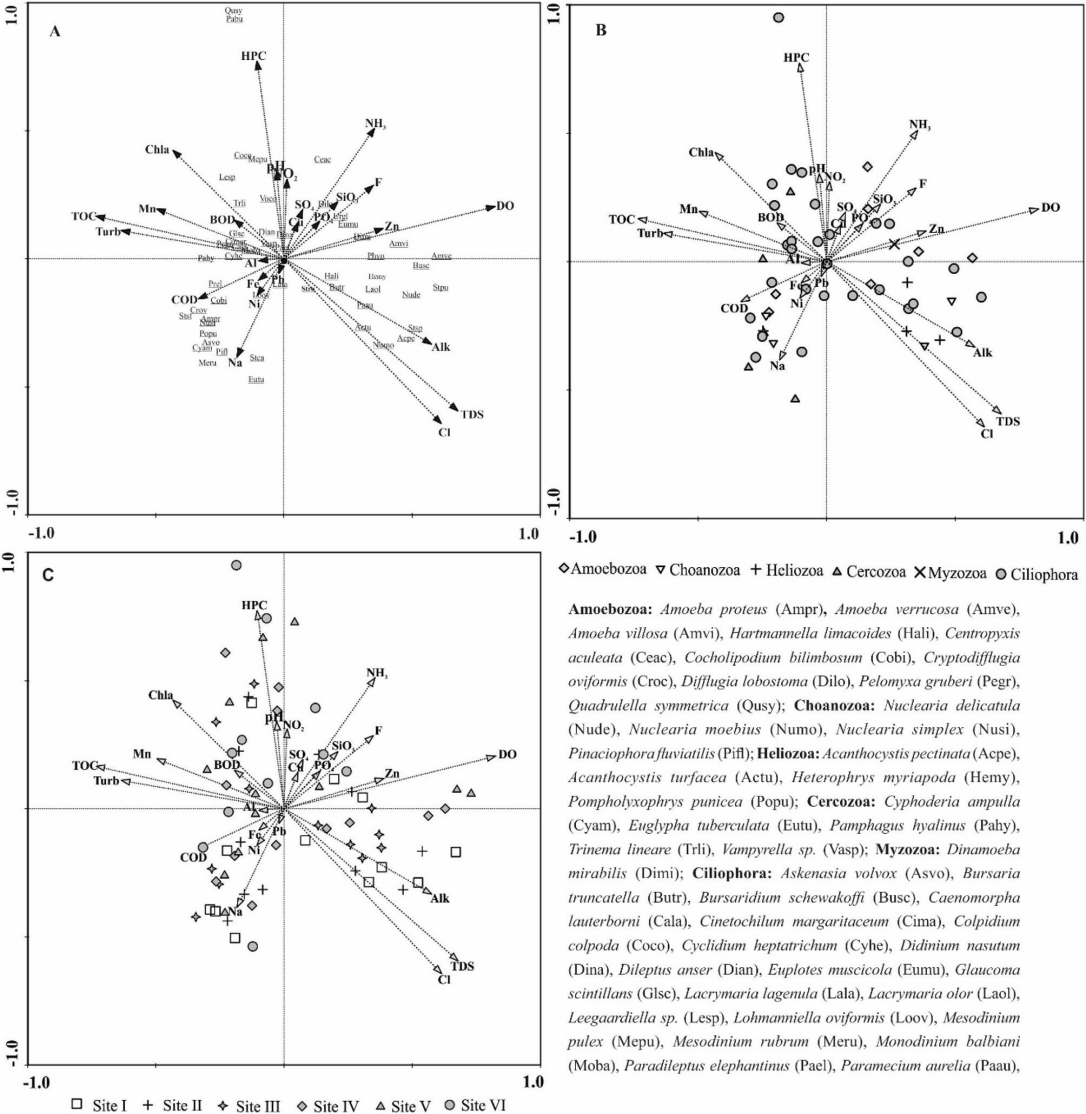
(**A**) Ordination diagram by CCA analysis of protozoa species as a function of environmental variables, (**B**) Systematic classification of species as a function of environmental variables. (**C**) Distribution of sampling units along the gradients of environmental variables. For abbreviations of environmental factors, see Table 2.

According to Spearman correlation, 26 variables were included in the CCA analysis, and six were eliminated. Temperature, EC, Ca^2+^, Mg^2+^, K^+^, and total hardness were strongly correlated and hence were excluded from ordination analysis. The CCA ordination model was significant in the first ( *P = 0.032*) and in all canonical axes (*P = 0.004* ). The eigenvalue of the first axis amounted 0.54, and for the second axis—0.34. These two axes captured 45.1% of the total variation of species data. On the CCA biplots, Protozoa communities of the sampling sites and species are visibly separated (Fig. 7A–C)). Axis1 correlated mostly with TDS, Cl, alkalinity, DO, NH_3_, and SiO_3_, while Axis2 correlated mostly with HPC, phytoplankton biomass, TOC, BOD, and turbidity.

The species of Heliozoa were associated mainly with the rising gradients of TDS, Cl, alkalinity, and DO. Leonov^79^ found Heliozoa to be a major component in water bodies with high mineralization and electric conductivity values. The growth of Heliozoa, among other groups, mostly benefited from the effect of dissolved salts, which facilitate their proliferation^80^. Compared with other sites, the high abundance of heliozoans at sites I and II may indicate their preference for clear water conditions.

The ameobozoans, including the most abundant, *Amoeba villosa* and *Centropyxis aculeata* were associated more with runoff variables. *Centropexis aculeata* appeared frequently only at the most stressed site, site VI. According to Roe and Patterson^81^, *C. aculeata* can colonize contaminated sites unfavourably to other species. Also, it is interesting to find the ameobozoan *Difflugia* between the vectors of ammonia and silicate. The species of this genus agglutinate particles from the environment to build their test and prefer to live in peatlands, likely because of the abundance of silica particles used for tests built^82^.

The cercozoans aggregated at the organic gradients, and the recorded species were found close to the vectors of TOC, COD, and phytoplankton biomass, indicating a great affinity of species for organic matter. Grazing experiments on Cercozoa demonstrated the indirect impact on the composition and function of bacteria in organic materials^83^. Also, Bass, et al.^84^ found that cercozoans adapted to anaerobic habitats with rich organic mud.

Ciliates are well-adapted to different environments, resulting in a higher richness and wide distribution range in the study area. The high species richness in this group is not surprising, given that it is a common planktonic taxon widely distributed in all or most habitats^85^. About 50% of ciliates, including the dominant bacterivorous, *Colpidium colpoda*, *Glaucoma scintillans*, and *Vorticella convallaria* correspond with the organic vectors of HPC, phytoplankton biomass and TOC of Axis2 (Fig. 7A). Ciliated Protozoa usually show dominance in eutrophic regions with more food sources, such as bacteria^86–88^, algae and organic matter^12,89^. Along Axis 1 that correlated with dissolved salts relevant variables, about 27% of ciliates species such as *Bursaridium schewakoffi*, *Strobilidium spiralis*, and *Stylonychia pustulata* were associated with one or more parameters like TDS, Cl, Do, and alkalinity. The most predominant *Paradileptus elephantinus*, *Didinium nasutum,* and *Monodinium balbiani* found to mediate the CCA environmental vectors, indicating that they exhibit no habitat type relationship, due to their high tolerance in addition to being omnivorous and predator species^28^.

### The saprobic index

**Figure 8:**
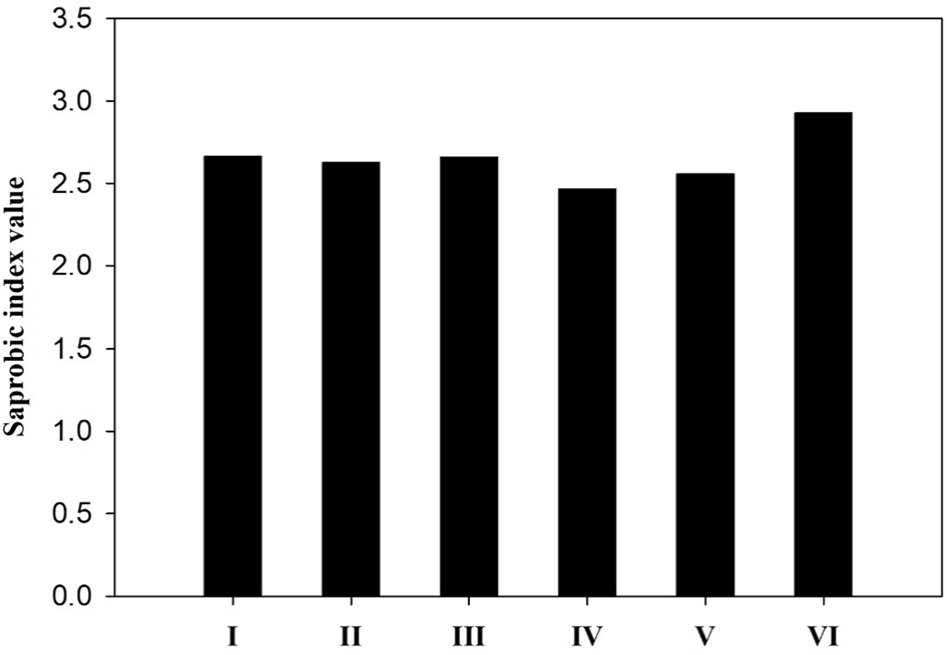
Mean saprobic index values at the six sampling sites.

The saprobic system is a biological monitoring method used for assessing organic pollution based on the different adaptabilities of protozoans in various fluvial water habitats^90^. Among the Protozoa species found in the Damietta branch, only 22 ciliates and five ameobozoans are included in the saprobic system (Table 4). Amoebozoa^91,92^ and Ciliophora^28,93^ are major groups of Protozoa widely used in saprobic water quality evaluation. The method is widely used to determine the degree of water pollution for evaluating water quality in rivers in many countries^12,34,94–96^. The saprobic index values (Fig. 8) ranged from 2.46 (site IV) to 2.92 (site VI), corresponding to class II-III and class III, respectively. The high SI value at sit VI (class III) indicated that the runoff input into the upstream was highly polluted with high levels of bacteria and organic pollutants. Organic pollutants cause an increase in nutrients, altering the structure of bacteria communities and inducing changes in bacterivorus ciliates^97^. In the present study, bacterivorus ciliates were particularly abundant (Table 4). *Caenomorpha lauterborni*, *Cinetochilum margaritaceum*, *Colpidium colpoda*, and *Glaucoma scintillans* are bacterivorous polysaprobic ciliates^28^; among them, some feed on sulfurous bacteria that are abundant in highly polluted water^12,98^ such as *Caenomorpha* that observed only at site VI.

## Conclusion

In summary, our results of the physicochemical parameters, as well as the multivariate analysis on the composition and structure of protozoans, showed a clear influence of anthropogenic disturbances in this part of the Nile River. Qualitative and quantitative analysis results have shed important light on the spatial and seasonal changes in Protozoa species diversity, abundance, and biomass. The patterns of Protozoa communities were significantly correlated with certain water quality parameter changes, especially the combination of TDS, PO_4_, NH_3_, NO_2_, DO, BOD, and TOC. The water quality parameters can be inferred by the presence of specifically identified protozoan species, suggesting the use of the bacterivorous ciliates *Colpidium campylum*, *Glaucoma scintillan*, and *Vorticella convallaria* as indicators of organic pollution, while *Strobilidium spiralis* and *Stylonychia pustulata* appear to reflect cleaner water. Ameobozoa species such as *Centropyxis aculeata* and *Amoeba villosa* can be used to indicate environments exposed to agricultural runoffs. The disappearance of the dominant Heliozoa species *Acanthocystis turfacea* can infer pollution induced by municipal drains. In addition, saprobic evaluations showed β- Mesosaprobic to α- mesosaprobic conditions at all sites except site VI, which showed an α-Mesosaprobic environment, revealing its higher organic pollution. These findings suggested that Protozoa can be employed as bioindicators in riverine waters affected by different degrees of pollution through their sensitivity to environmental parameters and contaminants.

## Data Availability

The datasets used and analyzed during the current study are available from the corresponding author upon reasonable request.

## References

[1] Negm AM (2017). The Nile River.

[2] Mohamed ZA, El-Sharouny HM, Ali WSM (2006). Microcystin production in benthic mats of cyanobacteria in the Nile River and irrigation canals, Egypt. Toxicon.

[3] Hassan FA (2005). A river runs through Egypt: Nile floods and civilization. Geotimes.

[4] Radwan TM, Blackburn GA, Whyatt JD, Atkinson PM (2019). Dramatic loss of agricultural land due to urban expansion threatens food security in the Nile Delta, Egypt. Remote Sens..

[5] Taher MES, Ghoneium AM, Hopcroft RR, ElTohamy WS (2021). Temporal and spatial variations of surface water quality in the Nile River of Damietta Region, Egypt. Environ. Monit. Assess..

[6] El-Tohamy WS, Hopcroft RR, Abdel Aziz NEM (2018). Environmental determinants of zooplankton community in the damietta estuary of the Nile River. Egypt. Pak. J. Zool..

[7] Asif N, Malik M, Chaudhry FN (2018). A review of on environmental pollution bioindicators. Pollution.

[8] Henebry MS, Ridgeway BT (1979). Epizoic ciliated Protozoa of planktonic copepods and cladocerans and their possible use as indicators of organic water pollution. Trans. Am. Microsc. Soc..

[9] Beaver JR, Crisman TL (1989). The role of ciliated protozoa in pelagic freshwater ecosystems. Microb. Ecol..

[10] Bonkowski M, Geoghegan IE, Birch ANSE, Griffiths BS (2001). Effects of soil decomposer invertebrates (Protozoa and earthworms) on an above ground phytophagous insect (cereal aphid) mediated through changes in the host plant. Oikos.

[11] Xu M (2005). Use of PFU protozoan community structural and functional characteristics in assessment of water quality in a large, highly polluted freshwater lake in China. J. Environ. Monit. Assess..

[12] Dias RJP, Wieloch AH, D'Agosto M (2008). The influence of environmental characteristics on the distribution of ciliates (Protozoa, Ciliophora) in an urban stream of southeast Brazil. Braz. J. Biol..

[13] Anderson OR (2010). Encyclopedia of Life Sciences (els).

[14] Debastiani C, Meira BR, Lansac-Tôha FM, Velho LFM, Lansac-Tôha FA (2016). Protozoa ciliates community structure in urban streams and their environmental use as indicators. Braz. J. Biol..

[15] Xu H, Zhang W, Jiang Y, Yang EJ (2014). Use of biofilm-dwelling ciliate communities to determine environmental quality status of coastal waters. Sci. Total Environ..

[16] Babko R (2020). Assessment of wastewater treatment plant effluent impact on the ecosystem of the river on the basis of the quantitative development of ciliated Protozoa characteristic of the aeration tank. Water Sci. Technol..

[17] Littleford RA (1960). Culture of protozoa in the classroom. Am. Biol. Teach..

[18] Galal MA, Khallaf E, Nabet N (2017). Ecological evaluation of ciliated Protozoa at Bahr-Shebeen canal, El-Menoufeyia province, Egypt. J. Biosci. Appl. Res..

[19] Corliss JO (2016). The Ciliated Protozoa: Characterization, Classification and Guide to the Literature.

[20] Galal MA (2018). Field studies on the protozoan distribution in Damietta and Rosetta branches of the River Nile, Egypt. J. Egypt. Acad. Soc. Environ. Dev. D Environ. Stud..

[21] Mola HRA, Ahmed NAM (2015). Zooplankton community structure and diversity relative to environmental variables in the River Nile from Helwan to El-Qanater El-Khayria, Egypt. Int. J. Environ..

[22] Eaton AD, Clesceri LS, Eugene WR, Greenberg AE (2005). Standard Methods for the Examination of Water and Wastewater.

[23] Sheen RT, Kahler HL, Ross EM, Betz WH, Betz LD (1935). Turbidimetric determination of sulfate in water. Ind. Eng. Chem. Anal. Edn..

[24] Wetzel RG, Likens GE (2013). Limnological Analyses.

[25] Shi X, Liu X, Liu G, Sun Z, Xu H (2012). An approach to analyzing spatial patterns of protozoan communities for assessing water quality in the Hangzhou section of Jing-Hang Grand Canal in China. Environ. Sci. Pollut. Res..

[26] Edmondson WT (1959). Freshwater Biology.

[27] Corliss JO (1979). The Ciliated Protozoa: Characterization, Classification, and Guide to the Literature.

[28] Foissner W, Berger H (1996). A user-friendly guide to the ciliates (Protozoa, Ciliophora) commonly used by hydrobiologists as bioindicators in rivers, lakes, and waste waters, with notes on their ecology. Freshw. Biol..

[29] Patterson DJ (1996). Freeliving Freshwater Protozoa.

[30] Gates MA, Rogerson A, Berger J (1982). Dry to wet weight biomass conversion constant for *Tetrahymena elliotti* (Ciliophora, Protozoa). Oecologia.

[31] Peet RK (1974). The measurement of species diversity. Annu. Rev. Ecol. Syst..

[32] Xu Z, Chen Y (1989). Aggregated intensity of dominant species of zooplankton in autumn in the East China Sea and Yellow Sea. Chin. J. Ecol..

[33] Pantle, R. & Buck, H. Die biologisch Uberwachung der Gewasser und die Darstellung der Ergebnisse. Gas-u. 604–624 (Gas und Wasserfach, 1955).

[34] Madoni P (2005). Ciliated protozoan communities and saprobic evaluation of water quality in the hilly zone of some tributaries of the Po River (northern Italy). Hydrobiologia.

[35] Revelle, W. R. psych: Procedures for personality and psychological research. *Available at: *http://CRAN.R-project.org/package=psych*.* (2017).

[36] Sousa W, Attayde JL, Rocha EDS, Eskinazi-Sant'Anna EM (2008). The response of zooplankton assemblages to variations in the water quality of four man-made lakes in semi-arid northeastern Brazil. J. Plankton Res..

[37] Cottenie K, Nuytten N, Michels E, De Meester L (2001). Zooplankton community structure and environmental conditions in a set of interconnected ponds. Hydrobiologia.

[38] Koutsikos N (2021). Defining non-indigenous fish assemblage types in Mediterranean rivers: Network analysis and management implications. J. Environ. Manag..

[39] Jongman RHG, Ter Braak CJF, Van Tongeren OFR (1995). Data Analysis in Community and Landscape Ecology.

[40] Ter Braak, C. J. F. & Smilauer, P. CANOCO reference manual and CanoDraw for Windows user's guide: software for canonical community ordination (version 4.5). (www. canoco. com, 2002).

[41] Ward JR (1963). Hierarchical grouping to optimize an objective function. J. Am. Stat. Assoc..

[42] Pal M, Nihar RS, Malabika BR, Pankaj KR (2015). Water quality index as a reliable indicator of water pollution level-A case study of Rudrasagar Lake, Tripura. Int. J. Innov. Res. Sci. Eng. Technol..

[43] World Health Organization (2003). Nitrate and nitrite in drinking-water: Background document for development of WHO Guidelines for Drinking-water Quality.

[44] Holm HW, Smith FA (1973). Effects of Protozoa on the Fate of Particulate Carbon.

[45] Michaud JP (1991). A Citizien's Guide to Understanding and Monitoring Lakes and Streams.

[46] Neori A, Holm-Hansen O (1982). Effect of temperature on rate of photosynthesis in Antarctic phytoplankton. Polar Biol..

[47] Atieh MA, Ji Y, Kochkodan V (2017). Metals in the environment: Toxic metals removal. Bioinorg. Chem. Appl..

[48] El-Geziry TM (2021). Monthly and annual variations in the rainfall pattern along the Southern Levantine Coastline. Res. Mar. Sci..

[49] Kotti ME, Vlessidis AG, Thanasoulias NC, Evmiridis NP (2005). Assessment of river water quality in Northwestern Greece. Water Resour. Manag..

[50] Kämäri M, Tattari S, Lotsari E, Koskiaho J, Lloyd CEM (2018). High-frequency monitoring reveals seasonal and event-scale water quality variation in a temporally frozen river. J. Hydrol..

[51] Parker JG (1983). Ciliated Protozoa in marine pollution studies: a conspectus. Ecotoxicol. Environ. Saf..

[52] Abo-Taleb HA, Abdel Aziz NE, Aboul Ezz SM, El Raey M, Abou Zaid MM (2016). Study of chromista and protozoa in a hotspot area at the Mediterranean coast with special reference to the potentiality to use it as bio-indicators. Int. J. Mar. Sci..

[53] Galal MA (2018). Protozoan diversity at certain provinces on the River Nile, Upper Egypt. J. Egypt. Acad. Soc. Environ. Dev. D Environ. Stud..

[54] Tan YH (2010). The relationships between ciliate composition, abundance, and environmental factors in Sanya Bay coral reef waters. Acta Ecol. Sin..

[55] Mansano AS, Hisatugo KF, Leite MA, Luzia AP, Regali-Seleghim MH (2013). Seasonal variation of the protozooplanktonic community in a tropical oligotrophic environment (Ilha Solteira reservoir, Brazil). Brazil. J. Biol..

[56] Brown RL, Reilly LAJ, Peet RK (2016). Encyclopedia of Life Science (els).

[57] Preston FW (1948). The commonness, and rarity, of species. Ecology.

[58] Fishar MR, Mahmoud NH, El-Feqy FA, Gaber KMG (2019). Community composition of zooplankton in El-Rayah El-Behery, Egypt. Egypt. J. Aquat. Biol. Fish..

[59] Bienert RW, Beaver JR, Crisman TL (1991). The contribution of ciliated protozoa to zooplankton biomass in an acidic, subtropical lake. J. Protozool..

[60] Meira BR (2017). Abundance and size structure of planktonic protist communities in a Neotropical floodplain: Effects of top-down and bottom-up controls. Acta Limnol. Brasiliensia.

[61] Chiarucci A, Wilson JB, Anderson BJ, De Dominicis V (1999). Cover versus biomass as an estimate of species abundance: Does it make a difference to the conclusions?. J. Veg. Sci..

[62] Dorgham MM, Abdel-Aziz EN, El-Ghobashy EA, El-Tohamy SW (2009). Preliminary study on protozoan community in Damietta Harbor, Egypt. Glob. Vet..

[63] Galotti A, Finlay BJ, Jiménez-Gómez F, Guerrero F, Esteban GF (2014). Most ciliated Protozoa in extreme environments are cryptic in the ‘seed bank’. Aquat. Microb. Ecol..

[64] Macek M, Šimek K, Bittl T (2001). Conspicuous peak of oligotrichous ciliates following winter stratification in a bog lake. J. Plankton Res..

[65] Caron DA, Goldman JC, Dennett MR (1986). Effect of temperature on growth, respiration, and nutrient regeneration by an omnivorous microflagellate. Appl. Environ. Microbiol..

[66] Duarte CM, Agustí S, Gasol JM, Vaqué D, Vazquez-Dominguez E (2000). Effect of nutrient supply on the biomass structure of planktonic communities: An experimental test on a Mediterranean coastal community. Mar. Ecol. Progress Ser..

[67] Crawford DW (2003). Influence of zinc and iron enrichments on phytoplankton growth in the northeastern subarctic Pacific. Limnol. Oceanogr..

[68] Nosek JN, Bereczky MC (1994). The effect of some environmental factors on protozoa populations of the River Danube. TISCIA Ecol. J. (Hungary).

[69] Thorp JH, Covich A (2001). Ecology and Classification of North American Freshwater Invertebrates.

[70] White, D., Johnston, K. & Miller, M. In: MD. Delong, TD. Jardine, AC. Benke, CE. Cushing (eds) *Rivers of North America (Second Edition)* 362–408 (Academic Press, 2023).

[71] Gensemer RW, Playle RC (1999). The bioavailability and toxicity of aluminium in aquatic environments. Crit. Rev. Environ. Sci. Technol..

[72] Sauvant MP, Pepin D, Bohatier J, Groliere CA (2000). Effects of chelators on the acute toxicity and bioavailability of aluminium to Tetrahymena pyriformis. Aquat. Toxicol..

[73] Abraham JV, Butler RD, Sigee DC (1997). Quantified elemental changes in Aspidisca cicada and Vorticella convallaria after exposure to aluminium, copper, and zinc. Protoplasma.

[74] Wantland WW (1956). Effect of various concentrations of sodium fluoride on parasitic and free-living protozoa and rotifera. J. Dent. Res..

[75] Šimek K (2019). Microbial food webs in hypertrophic fishponds: Omnivorous ciliate taxa are major protistan bacterivores. Limnol. Oceanogr..

[76] Jiang Y (2011). An approach to analyzing spatial patterns of planktonic ciliate communities for monitoring water quality in Jiaozhou Bay, northern China. Mar. Pollut. Bull..

[77] Rakshit D (2017). Bioindicator role of tintinnid (Protozoa: Ciliophora) for water quality monitoring in Kalpakkam, Tamil Nadu, south east coast of India. Mar. Pollut. Bull..

[78] Xu H, Jiang Y, Al-Rasheid KA, Al-Farraj SA, Song W (2011). Application of an indicator based on taxonomic relatedness of ciliated protozoan assemblages for marine environmental assessment. Environ. Sci. Pollut. Res..

[79] Leonov MM (2010). Heliozoans (Heliozoa, Sarcodina, Protista) of fresh and marine waters of the European part of Russia: Species composition, morphology, and distribution. Inland Water Biol..

[80] Sente C (2016). Prevalence of pathogenic free-living amoeba and other protozoa in natural and communal piped tap water from Queen Elizabeth protected area, Uganda. Infect. Dis. Poverty.

[81] Roe HM, Patterson RT (2006). Distribution of thecamoebians (testate amoebae) in small lakes and ponds, Barbados, West Indies. J. Foraminifer. Res..

[82] Mitchell EAD, Charman DJ, Warner BG (2008). Testate amoebae analysis in ecological and paleoecological studies of wetlands: Past, present and future. Biodivers. Conserv..

[83] Flues S, Bass D, Bonkowski M (2017). Grazing of leaf-associated Cercomonads (Protists: Rhizaria: Cercozoa) structures bacterial community composition and function. Environ. Microbial..

[84] Bass D (2009). Phylogeny of novel naked filose and reticulose Cercozoa: Granofilosea cl. N. and Proteomyxidea revised. Protist.

[85] Liu W (2021). Distribution patterns of ciliate diversity in the South China Sea. Front. Microbiol..

[86] Albright LJ, Sherr EB, Sherr BF, Fallon RD (1987). Grazing of ciliated protozoa on free and particle-attached bacteria. Mar. Ecol. Progress Ser..

[87] Amblard C, Sime-Ngando T, Rachiq S, Bourdier G (1993). Importance of ciliated protozoa in relation to the bacterial and phytoplanktonic biomass in an oligo-mesotrophic lake, during the spring diatom bloom. Aquat. Sci..

[88] Shukla U, Gupta PK (2001). Assemblage of ciliated protozoan community in a polluted and non-polluted environment in a tropical lake of central Himalaya: Lake Naini Tal, India. J. Plankton Res..

[89] Mieczan T (2007). Relationships among ciliated Protozoa and water chemistry in small peat-bog reservoirs (Łęczna-Włodawa Lakeland, Eastern Poland). Oceanol. Hydrobiol. Stud..

[90] Chen Q-H, Xu R-L, Tam NFY, Cheung SG, Shin PKS (2008). Use of ciliates (Protozoa: Ciliophora) as bioindicator to assess sediment quality of two constructed mangrove sewage treatment belts in Southern China. Mar. Pollut. Bull..

[91] Chantangsi C (2001). An investigation on protozoan diversity in α-mesosaprobic zone of a man-made canal at Chulalongkorn University. Trop. Nat. Hist..

[92] Opravilová V (1986). Testacea (Protozoa: rhizopoda) in the epilithion of the lotic stretch of running waters of different degrees of saprobity (Czechoslovakia). Acta Hydrochimica Et Hydrobiologica.

[93] Foissner, W. *Taxonomische und ökologische Revision der Ciliaten des Saprobiensystems: Cyrtophorida, Oligotrichida, Hypotrichia, Colpodea*. Vol. 91 (Bayerisches Landesamt für Wasserwirtschaft, 1991).

[94] Komala, R. & Nurfitriana, D. In: *IOP Conference Series: Materials Science and Engineering.* 052057 (IOP Publishing).

[95] Madoni P, Braghiroli S (2007). Changes in the ciliate assemblage along a fluvial system related to physical, chemical and geomorphological characteristics. Eur. J. Protistol..

[96] Walley WJ, Grbović J, Džeroski S (2001). A reappraisal of saprobic values and indicator weights based on Slovenian river quality data. Water Res..

[97] Primc B (1988). Trophic relationships of ciliated Protozoa developed under different saprobic conditions in the periphyton of the Sava River. Periodicum Biologorum.

[98] Czapik A (1982). The effect of waste water on ciliate communities in the Biata Przemsza River. Acta Hydrobiologica.

